# Cognition in adults with bottom‐of‐sulcus dysplasia and the consequences of focal resection

**DOI:** 10.1002/epi.70116

**Published:** 2026-02-05

**Authors:** Carmen J. Zheng, David Weintrob, Marie O'Shea, Graeme D. Jackson, Chris Tailby

**Affiliations:** ^1^ Florey Department of Neuroscience and Mental Health University of Melbourne Parkville Victoria Australia; ^2^ Florey Institute of Neuroscience and Mental Health Heidelberg Victoria Australia; ^3^ Department of Psychology Austin Health Heidelberg Victoria Australia; ^4^ Melbourne School of Psychological Sciences University of Melbourne Parkville Victoria Australia; ^5^ Department of Neurology Austin Health Heidelberg Victoria Australia

**Keywords:** age at seizure onset, cortical malformation, focal cortical dysplasia, focal epilepsy, postsurgical outcomes

## Abstract

**Objective:**

To determine whether there are cognitive consequences of bottom‐of‐sulcus dysplasia (BOSD) when assessed as adults and whether focal resection of these lesions leads to change in cognition.

**Methods:**

We studied 42 adults, of whom 39 underwent focal resection targeting the lesion. Neuropsychological assessments were tailored to the clinical epileptology.

**Results:**

On average, patients were 30 (15–56) years old at the time of assessment. Epilepsy duration was 21 (3–48) years. Seizure onset ranged between 3 months and 26 years. Fifteen patients (36%) had an age of onset of 12 years or older. Older age of seizure onset correlated with fewer cognitive measures impacted (*r* = −0.34, *p* = .026). Frontal (52%) and parietal (33%) lobes were common BOSD locations. Confrontation naming was assessed in 20 patients, 11 (55%) of whom were impaired. Of these 11 patients, 8 (73%) had a left‐sided BOSD. Verbal fluency was assessed in 23 patients, 13 (57%) of whom were impaired. Of these 13 patients, 11 (85%) had a frontal BOSD. Processing speed and attention were assessed in 35 patients and deficits were seen in 17% to 20%, though milder reductions were more consistently seen. At post‐surgical follow‐up (*M* = 8.07 years, SD = 4.91 years), 59% of patients were seizure free. As a group, there was no evidence of post‐surgical cognitive decline after focal resection of the BOSD; processing speed (*p* < .05) improved post‐surgically.

**Significance:**

In adults with BOSD, an earlier age of seizure onset is accompanied by a greater degree of cognitive comorbidity. Naming is commonly affected, particularly for those with left sided BOSDs. Executive dysfunction is common, particularly for patients with a frontal BOSD. Focal lesionectomy is associated with favourable seizure outcome and is cognitively safe with potential for improvement in processing speed.

**Key points**

- Focal lesionectomy of BOSD is effective for seizure control and has low cognitive risk.
- Deficits in naming and executive function are frequently seen in adults with BOSD.
- Younger age at seizure onset is associated with more cognitive comorbidity.
- BOSD resection is associated with improved processing speed.

## 1 INTRODUCTION

Focal cortical dysplasia (FCD) refers to a heterogeneous group of focal cortical malformation and a common cause of drug‐resistant epilepsy.^1^ In particular, FCD type II (FCDII) is characterized by abnormal cortical lamination with the presence of dysmorphic cells and is often easier to identify by imaging than other FCD types.^2^, ^3^, ^4^ Bottom‐of‐sulcus dysplasia (BOSD) is an important subtype of FCDII in which imaging and pathologic abnormalities are maximal in the depth of the affected sulcus.^5^ These lesions are highly epileptogenic, often associated with seizure onset in early childhood.^6^ Seizures in the setting of BOSDs are rarely adequately controlled by antiseizure medications (ASMs) alone.^7^ Surgical resection of the lesion often remains the only curative treatment, yielding favorable seizure outcomes.^8^, ^9^, ^10^, ^11^

The current literature on cognitive features of BOSD is mostly focused on children undergoing presurgical evaluation. In these cases, BOSD is associated with language and executive dysfunction, and intellectual disability (ID) is associated with earlier onset of epilepsy.^6^, ^12^ Recent evidence suggested that executive and naming deficits may be related to the frontal predilection of BOSD, overlapping with functional networks critical to these cognitive processes.^12^

Adults with BOSD assessed in our epilepsy surgery program have baseline cognitive testing prior to surgery. This allowed us to determine whether BOSD is associated with cognitive compromise in adults preoperatively and determine the extent that subsequent focal resection impacts cognitive performance. Given that BOSD is likely nonfunctional tissue^13^, ^14^ and exhibits continuous spiking associated with extreme epileptogenicity,^15^ we hypothesize that focal resection would be cognitively safe or even beneficial.^16^

## 2 MATERIALS AND METHODS

### 2.1 Patient ascertainment

We retrospectively identified 44 adults with BOSD‐associated drug‐resistant focal epilepsy, evaluated between 1992 and 2022 in the Bladin‐Berkovic Comprehensive Epilepsy Programme of Austin Health, Melbourne, Australia. BOSD diagnosis was based on magnetic resonance imaging (MRI) showing features including the following: cortical thickening and gray‐white matter blurring being maximal at the depth of the sulcus, increased subcortical T2 or fluid‐attenuated inversion recovery signal, and a transmantle band extending toward the ventricle.^4^, ^9^ One patient was excluded from the analysis due to previous epilepsy surgery and another due to additional co‐occurring malformations (i.e., hemimegalencephaly and extensive periventricular nodular heterotopia in the same hemisphere as the BOSD). Overall, this yielded a final sample of 42 patients, 39 of whom proceeded to surgery, with histological confirmation obtained in 38; the remaining patient underwent radiofrequency thermocoagulation. The surgical approach implemented in our program is targeted corticectomy limited to the dysplastic sulcus without extending below the gray–white matter junction, to minimize the risk of injury to white matter tracts.^9^

### 2.2 Cognitive data

Psychometric scores were obtained from cognitive assessments conducted by clinical neuropsychologists with extensive experience in epilepsy (D.W., M.O., C.T.). Acquisition of clinical history and psychometric testing were tailored to detect the presence of any potential cognitive deficits, considering the clinical context of each patient.

We identified 11 cognitive tests that were most consistently administered across the sample, categorizing each of these tasks to a specific cognitive domain (Table S1). All raw test scores were converted to age‐adjusted *z*‐scores based on normative data and administrative manuals prior to analysis.^17^, ^18^, ^19^, ^20^ We applied a threshold of 5th percentile (i.e., *z* < −1.645) as criterion of marked psychometric deficit.

### 2.3 Analysis of baseline and postsurgical cognition

Baseline cognition was defined as the first documented score for each cognitive test. For each cognitive measure, we first considered performance of the group as a whole, before evaluating effects of BOSD laterality (i.e., left vs. right hemisphere) and focality (i.e., frontal vs. parietal lobe only, considering rarity of BOSD in other lobes as described below).

We obtained presurgical cognitive scores from the assessment closest in time to surgery. For individuals who underwent BOSD resection, we obtained postsurgical scores from the earliest assessment occurring at least 60 days following the last surgery; postsurgical change for each cognitive test was compared longitudinally.

### 2.4 Clinical variables

Age at seizure onset, estimated seizure frequency per month, and the number of ASMs were retrieved from medical records closest to the date of when the cognitive score was attained, where available. We also reviewed all neuropsychological reports to note the presence of psychiatric comorbidities at the time of assessment, and whether this may have impacted patients' cognitive performance. Duration of epilepsy was calculated as the difference between age at seizure onset and the date of cognitive assessment. Postsurgical seizure outcomes and ASM counts were collected at 1‐year follow‐up and the most recent follow‐up thereafter, where available.

### 2.5 Data analysis

All statistical analyses were performed using R (version 4.4.1). Cognitive performances were analyzed in terms of *z* units. For baseline cognition, one‐sample *t*‐test was used to analyze normally distributed data, using Hedge *g* as a measure of effect size; Wilcoxon signed‐rank test (*W* statistic) was used to analyze nonnormally distributed data, using rank‐biserial correlation (*r*_*rb*_) as a measure of effect size. Between‐subjects comparisons (e.g., comparison by BOSD laterality or lobe) of cognitive scores were analyzed using Welch *t*‐test and Wilcoxon rank‐sum test for nonnormally distributed data. To measure within‐subjects postsurgical cognitive change, we calculated longitudinal difference in performance for each patient and across each domain. Normally distributed cognitive change scores were analyzed using one‐sample *t*‐test and Wilcoxon signed‐rank test for nonnormally distributed change scores. The relationship between each cognitive domain with respect to ASM count and age at onset was measured by Pearson correlation. All analyses were corrected for false discovery rate.^21^

## 3 RESULTS

### 3.1 Patient demographics

A total of 42 patients (20 females) were included in our study. Age at first seizure ranged from 3 months to 26 years (mean = 9.0 ± 6.5 years). Age at first neuropsychological assessment ranged between 15.4 and 55.9 years (mean = 29.8 ± 10.5). Duration of epilepsy at the time of baseline assessment averaged 20.7 ± 10.7 (range = 3.1–48.2) years. Average number of ASMs taken was 2.6 ± 1.1 (range = 1–5). Median seizure frequency per month was 37.5 (interquartile range [IQR] = 8–142.5, range = 0–4500; this includes one patient with 150 brief seizures recorded during 24 h of video‐electroencephalographic monitoring, extrapolated to 4500 per month).

Eighteen (42.9%) patients had a history of diagnoses or symptoms of depression, anxiety, psychosis, and/or antisocial and/or narcissistic personality traits. Review of the neuropsychological formulation in these 18 cases revealed that in only two patients was the psychiatric history felt to have potentially affected cognitive performance (i.e., generalized compromise of speed and attention). A total of seven (16.7%) patients presented with developmental dyslexia. In addition, one was noted to have Lennox–Gastaut syndrome.

For the three patients with ID, the age at first seizure was within the first 2 years (18, 21, 3 months, respectively). The first patient, with severe ID and comorbid autism spectrum disorder, was not formally assessed by our neuropsychological service. For the remaining two patients, duration of epilepsy at the time of assessment was 17.4 and 23.5 years; an ID was also present at moderate and mild severity, respectively.

**FIGURE 1:**
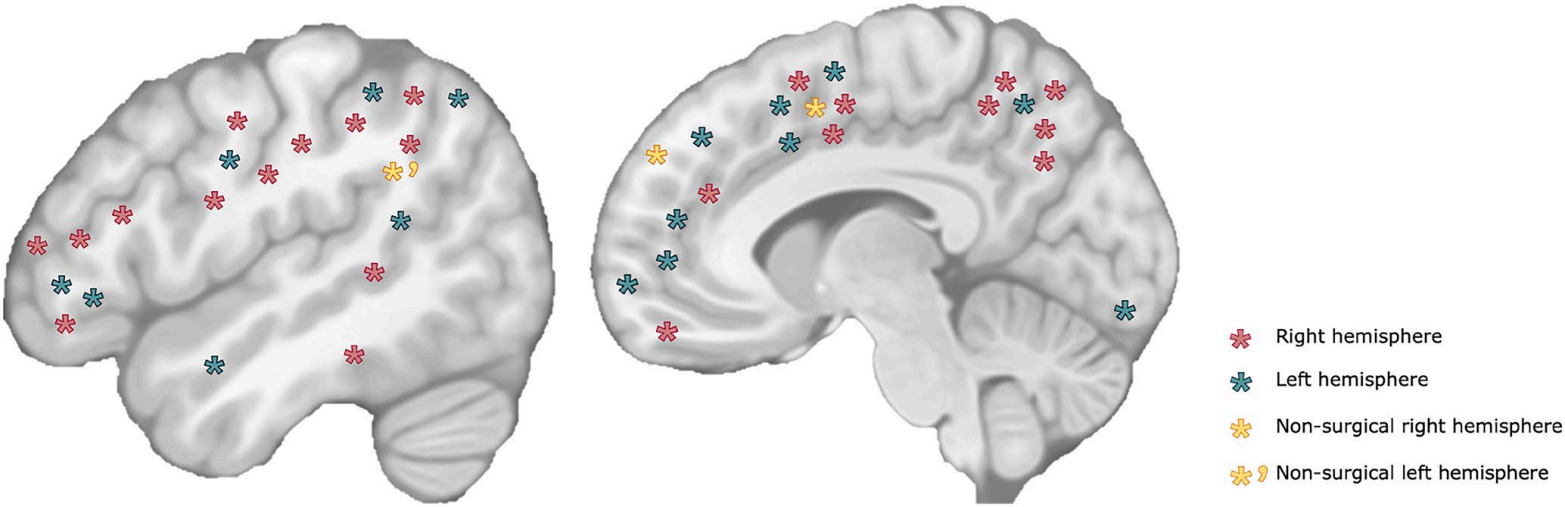
Localization and lateralization of bottom‐of‐sulcus dysplasias (BOSDs). Schematic diagram shows BOSD locations in the 42 patients. For those who underwent surgery, blue asterisks indicate left‐sided BOSD (*n* = 16), and red asterisks indicate right‐sided BOSD (*n* = 23). BOSD locations of patients who did not undergo surgery are shown as yellow asterisks (*n* = 3), with left‐sided BOSD denoted by an asterisk‐prime.

Across the cohort, 17 patients (40.5%) had a left‐sided BOSD (Figure 1). The most common lobar location of BOSD was frontal (22 cases, 52.4%), followed by parietal (14 cases, 33.3%).

### 3.2 Younger age at seizure onset correlated with greater counts of marked psychometric deficits

**FIGURE 2:**
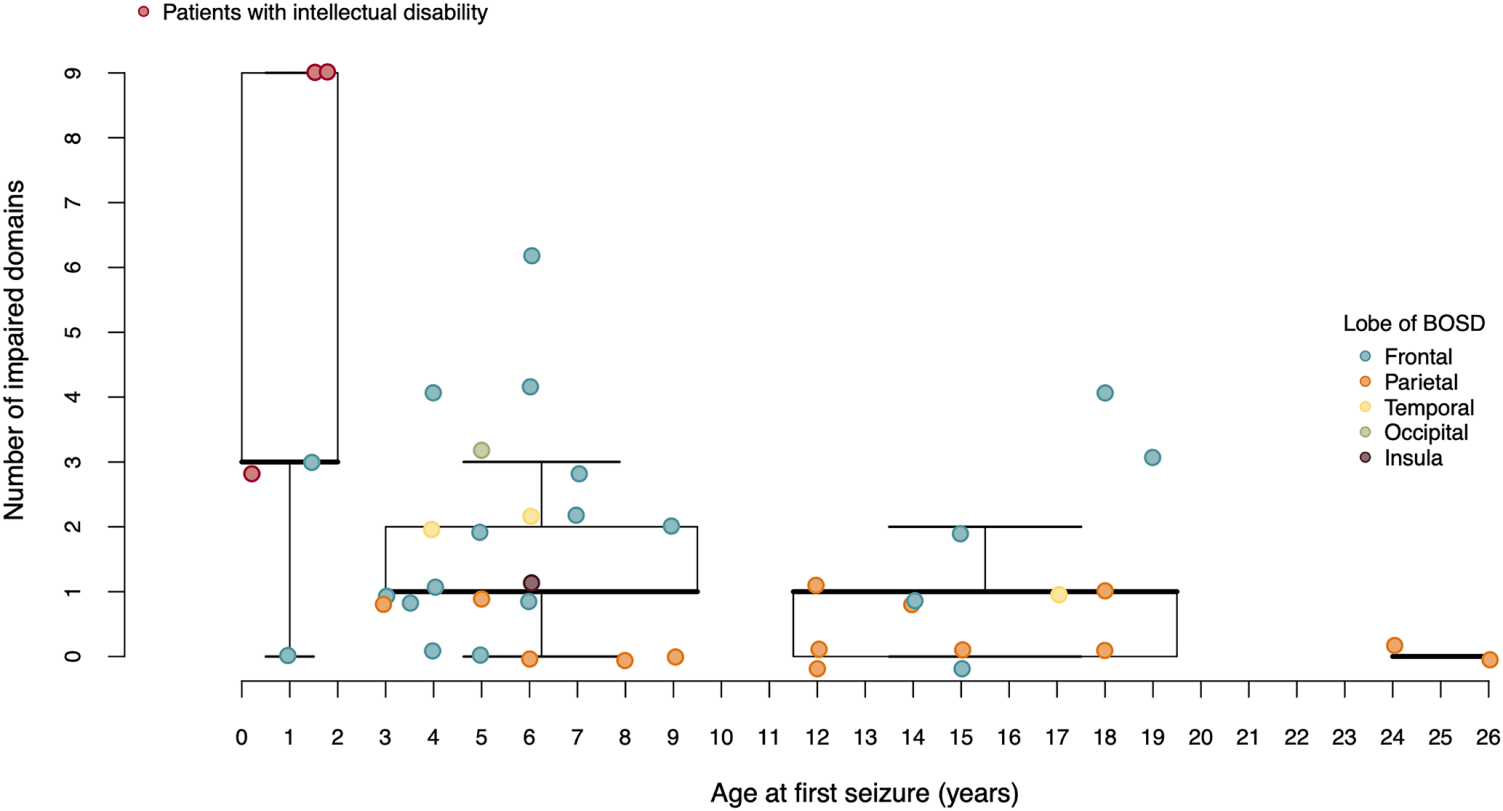
Number of cognitive tests showing marked psychometric deficits as a function of age at first seizure (years). Patients with intellectual disability (ID) are highlighted in red (*n* = 3). One case of severe ID was not assessed due to their level of cognitive impairment. For this case, we imputed the number of impaired cognitive tests as nine, matching the maximum number of impaired tests observed in patients for whom cognitive data were available. BOSD, bottom‐of‐sulcus dysplasia.

For each patient, we collated the number of test scores falling below the 5th percentile of normative expectations (i.e., marked psychometric deficits). We plotted these counts against the age at seizure onset (Figure 2).

Three of five patients with age at onset earlier than 3 years had an ID. Twenty‐two patients had an age at seizure onset between 3 and 10 years; their average count of marked psychometric deficits was 1.68 ± 1.55. Thirteen patients had an age at seizure onset between 10 and 20 years; their average count of marked psychometric deficits was 1.08 ± 1.26. Two patients with an age at seizure onset after 20 years had no psychometric deficits. There was a significant negative correlation (*r* = −.34, *p* = .026) between age at first seizure (when treated as a continuous variable) and the number of impaired test performances. No such relationship (*p* > .05) was found between duration of epilepsy, ASM count, or current seizure frequency and number of impaired test performances.

Patients with frontal BOSD had a significantly younger (*W* = 76, *p* = .012) age at first seizure (mean_[frontal]_ = 7.05 years) than patients with parietal BOSD (mean_[parietal]_ = 13 years), with a moderate effect size (*r*_*rb*_ = .42). Patients with frontal BOSD also had a significantly greater (*W* = 258, *p* < .001, *r*_*rb*_ = .58) number of impaired test scores (mean_[frontal]_ = 2.36) than those with parietal BOSD (mean_[parietal]_ = .36). This effect persisted after removing two measures (i.e., verbal fluency, working memory) that were administered more often in frontal cases (mean_[frontal]_ = 1.59, mean_[parietal]_ = .21; *W* = 234, *p* = .005), with a moderate effect size (*r*_*rb*_ = .47).

### 3.3 Group‐level mild to moderate reduction in speed, attention, and executive function

**FIGURE 3:**
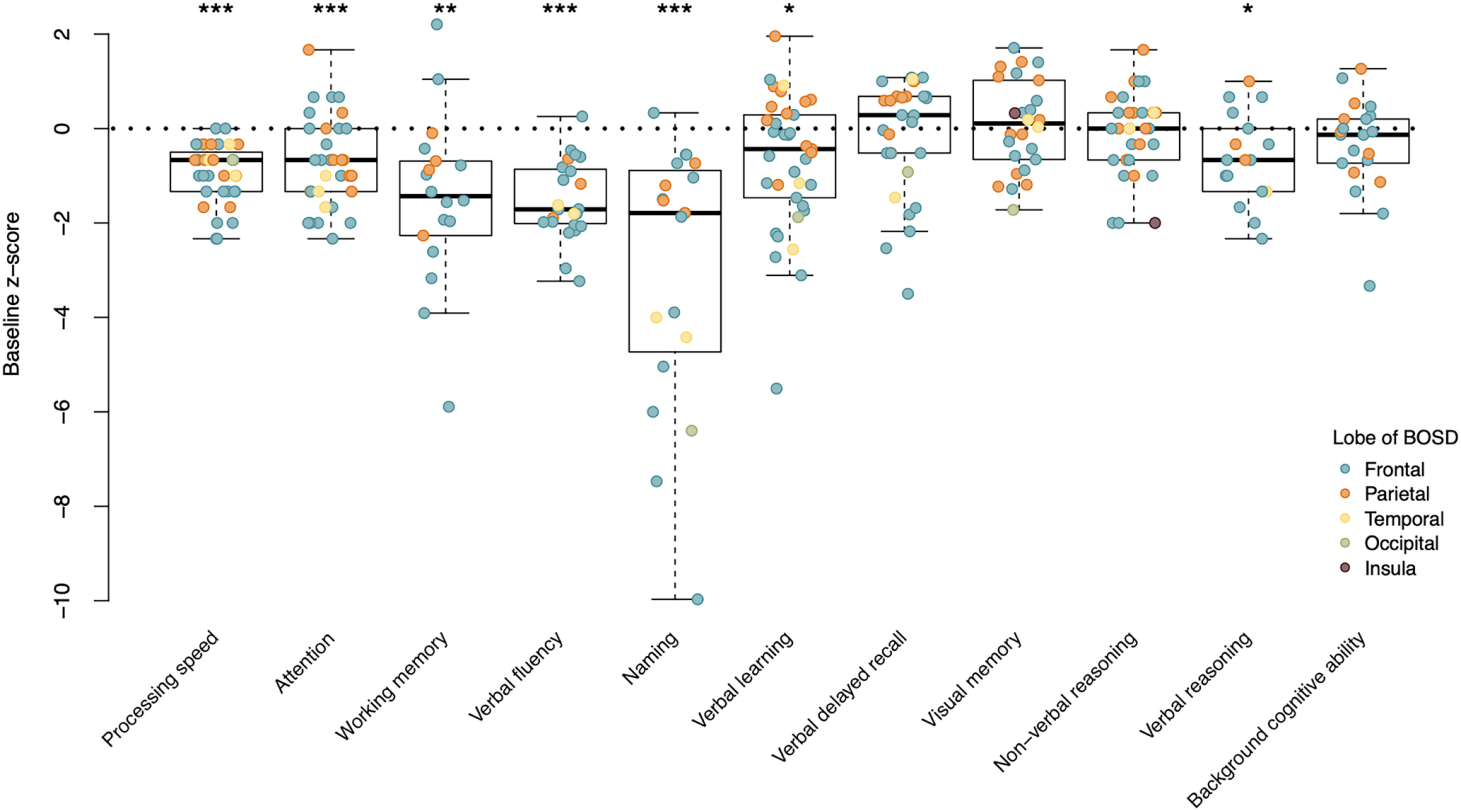
Boxplot of *z*‐scores for each cognitive test at baseline. Negative values correspond to performance below normative expectation. Statistical significance in one‐sample *t*‐test (relative to the expected mean of zero): *p < .05, **p < .01, ***p < .001. BOSD, bottom‐of‐sulcus dysplasia.

Figure 3 shows norm‐referenced *z*‐scores for each cognitive test at baseline (i.e., where there is no history of neurosurgery). At the group level, processing speed, attention, aspects of executive function (i.e., verbal fluency, working memory), and verbal reasoning were significantly below normative expectations. Confrontation naming, assessed more often in left hemisphere cases, fell a median of 1.79 *z*‐score units below normative expectation. Word list learning was .69 *z*‐score units below the norm, although delayed recall of that word list was comparable to age expectations. Visual memory, nonverbal reasoning, and background cognitive ability were also comparable with age expectations.

Our exploratory analyses showed ASM count negatively correlated with verbal fluency *z*‐score (*r* = −.50, *p* = .028); no other cognitive measures showed an association with ASM count.

### 3.4 Naming, verbal fluency, and working memory are the most commonly affected domains

**FIGURE 4:**
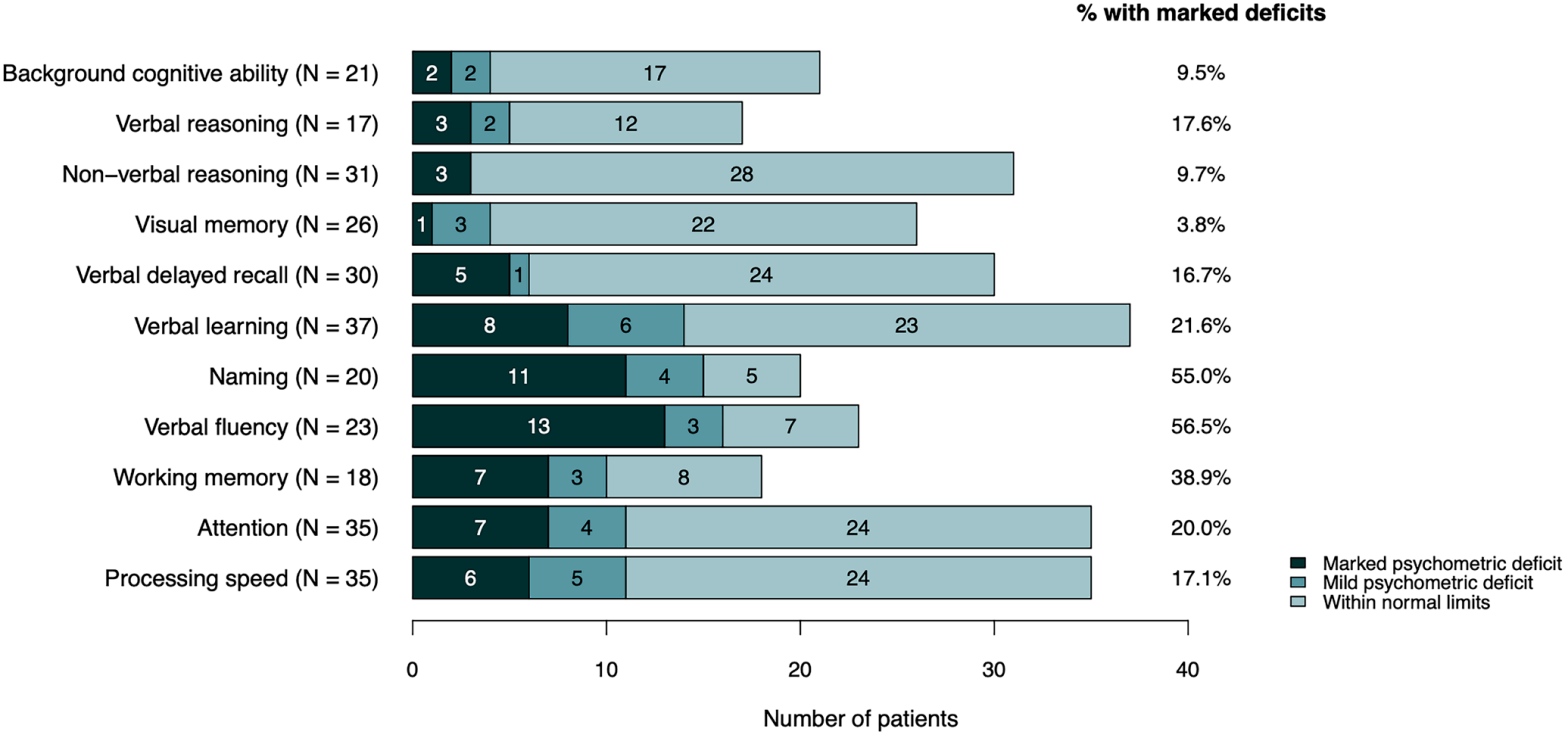
Frequency of cases showing marked psychometric deficits for each cognitive measure. Marked psychometric deficit = performances below the 5th percentile (i.e., *z*‐score < −1.645); mild psychometric deficit = performances between 5th and 15.9th percentile (i.e., −1.645 ≤ *z*‐score ≤ −1); within normal limits = performances above 15.9th percentile (i.e., *z*‐score ≥ −1). Total number of patients measured in each domain is indicated within parentheses, along with the proportion of patients showing marked psychometric deficit.

At the individual patient level, marked psychometric deficits were detected most frequently on measures of verbal fluency (56.5%), naming (55.0%), and working memory (38.9%; Figure 4). In particular, eight of 11 cases (72.7%) showing a naming impairment had a left‐sided BOSD, and 11 of 13 cases (84.6%) with impaired verbal fluency were frontal cases of BOSD.

The most commonly evaluated domain was verbal learning, with approximately one in five patients showing marked psychometric deficits in this sphere. Processing speed and attention were examined in 35 patients; a marked impairment of processing speed was present in 17% of these cases, and 20% exhibited an objectively defined attentional dysfunction.

### 3.5 Effects of frontal BOSD on cognition

In addition to group‐level analysis, we sought to consider whether there were any potential effects of BOSD laterality (i.e., left vs. right hemisphere) and focality (i.e., frontal vs. parietal lobe).

Among those cognitive tests with appropriately balanced sample sizes (i.e., there being no statistically significant bias between two sample sizes in a comparison; see Table S2), we found a significant difference in verbal learning between patients with frontal and parietal BOSD. Specifically, performance of the frontal (mean_[frontal]_ = −1.21) BOSD group was significantly lower than the parietal (mean_[parietal]_ = .27) BOSD group (*t*_[30.1]_ = −3.72, *p* = .009), with a large effect size (Hedge *g* = −1.14). To address potential impacts of having fewer left parietal cases (*n* = 4) than left frontal cases (*n* = 10) in this lobewise comparison—given that verbal learning is generally left lateralized—we further differentiated between cases of right‐sided frontal BOSD (*n* = 11) and right‐sided parietal BOSD (*n* = 9) tested for verbal memory. The result showed that patients with right‐sided frontal BOSD still performed significantly worse than right‐sided parietal BOSD in verbal learning (*t*_[17.73]_ = −4.26, *p* < .001, mean_[frontal]_ = −1.01, mean_[parietal]_ = .21), with a large effect size (Hedge *g* = −1.77).

### 3.6 Case vignette

**FIGURE 5:**
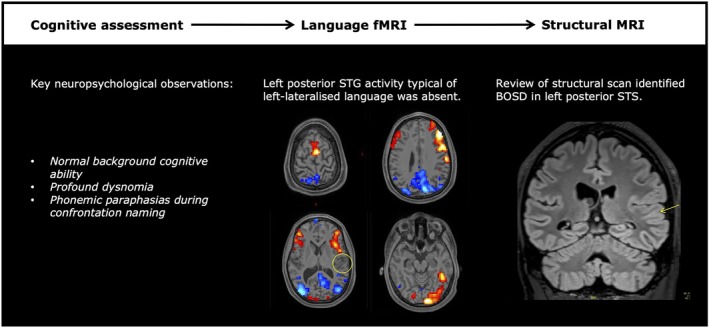
Illustrative case with bottom‐of‐sulcus dysplasia (BOSD) in left posterior superior temporal sulcus (STS). Neuropsychological findings were suggestive of left posterior perisylvian dysfunction. Language functional magnetic resonance imaging (fMRI) showed left lateralized activation, although this is notable for the absence of activation typically seen in posterior superior temporal cortex on this nonword rhyming task (yellow circle; feature threshold *p* < .001, familywise error‐corrected *cluster threshold p* < .05 two tailed), suggesting a functional abnormality in this area. On review of structural MRI, an unusual sulcus in the superior temporal gyrus (STG) with an appearance of the transmantle sign was identified, postsurgically confirmed as a focal cortical dysplasia type IIB.

As an illustrative case of BOSD, we describe neuropsychological findings in an 18‐year‐old left‐handed male with onset of sleep‐related hypermotor epilepsy at age 6 years. Multiple MRI studies undertaken since seizure onset had been reported as normal. His seizures were characterized by facial distortion, hand shaking, oral automatism, and postictal dysphasia. On neuropsychological assessment, background cognitive ability was normal, although there were features of a developmental dyslexia. This finding was accompanied by a dysnomia marked by phonemic paraphasias (evident on visual confrontation naming). Language functional MRI^22^ was left lateralized, although notable for the absence of activation typically seen in the left posterior superior temporal sulcus (Figure 5). This clinical neuropsychological profile suggested a left posterior temporal focus; a targeted review of the structural MRI disclosed a BOSD in left posterior superior temporal sulcus.

### 3.7 Postsurgical cognition and seizure control

A total of 39 patients underwent targeted lesionectomy of BOSD during the study period. Of these, five patients had a repeat resection of BOSD, and one patient underwent three separate resective procedures. Histology confirmed FCD in 37 patients, of whom 31 patients had FCDIIB and six had FCDIIA. In one patient, the available histology sample did not reveal features of FCDII, but the diagnosis was supported by a convincing MRI. Structural MRI of the second patient was reported as very suspicious of a BOSD in the depths of the anterior bank of the left central sulcus. Further investigations including functional MRI, fluorodeoxyglucose positron emission tomography, and ictal single photon emission computed tomography and a characteristic signature in this region on stereo‐electroencephalography converged to support localization of an FCD in this region, as reported recently.^23^ This patient underwent radiofrequency ablation; a histology sample was therefore not applicable.

**FIGURE 6:**
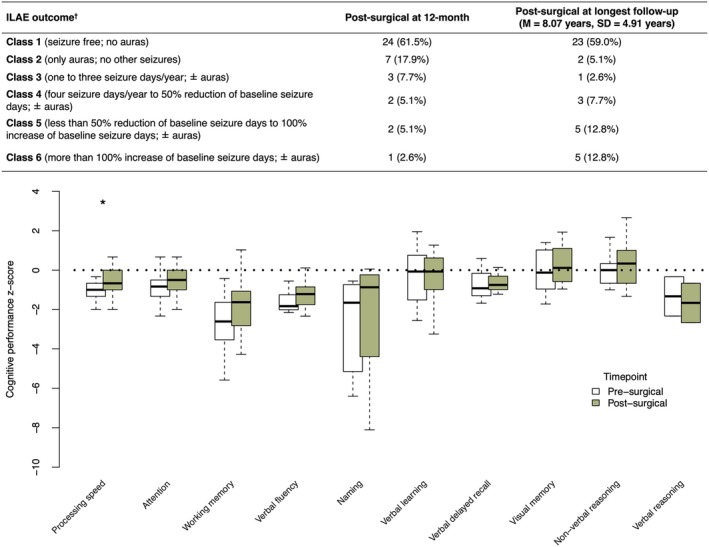
Postsurgical seizure and cognitive outcomes. Top panel shows postsurgical seizure outcomes at both 12 months and the most recent review. ^†^Postsurgical seizure outcome classified as per International League Against Epilepsy (ILAE) guidelines.^24^ Bottom panel shows longitudinal comparison of *z*‐scores for each cognitive domain at 3 months postsurgery compared to presurgical scores. Negative values correspond to performance lower than normative expectation. Significant postsurgical improvement was evident in processing speed longitudinally. Background cognitive ability was not assessed postsurgically. *p < .05. M, mean.

Data regarding seizure outcomes are reported in Figure 6 (top panel). More than sixty percent of the patients achieved seizure freedom at 12 months. At the most recent neurological review (mean = 8.07 years, SD = 4.91 years), 64% of patients had an International League Against Epilepsy (ILAE) outcome of class 2 or better. Five (13%) patients had a class 5 outcome, and five (13%) patients had a more than 100% increase of baseline seizure frequency at their longest follow‐up (ILAE outcome class 6). ASM counts generally remained stable postsurgically, with a modal count of two (range = 0–5).

Of the 39 patients who underwent surgery, 31 (80%) had pre‐ and postsurgical cognitive longitudinal data available. The median duration from presurgical assessment to first surgery was 213 (IQR = 117.5–399.5) days, the median duration from the latest surgery to postsurgical assessment was 96 (IQR = 89–104) days, and the median duration between pre‐ and postsurgical assessments was 395.73 (IQR = 231.84–818.94) days.

Boxplots of longitudinal comparison of pre‐ and postsurgical scores, in *z*‐units, are shown for each cognitive domain (Figure 6, bottom panel). Processing speed (*p* = .02, *r*_*rb*_ = .83) showed significant improvement postsurgically. The observed postsurgical improvement was consistent across all levels of presurgical performance, with 77% (17 of 22 patients assessed for processing speed) showing no change or an improvement in postsurgical performance. Of note, the improvement in processing speed occurred in the setting of stable or even increased number of ASMs taken in 82% of patients. Postsurgical performance in the remaining domains was stable or demonstrated a slight increment.

No postsurgical cognitive decline was observed on any measure at a group level. Four individual cases showed more than 1.5 *z*‐score units decline longitudinally. One case of left frontal BOSD, proximal to Broca area, showed a presurgical naming deficit (*z* = −6.0) in the setting of an ID. In this patient, naming worsened postoperatively (*z* = −8.1). Postsurgical imaging of this patient showed that the resection included adjacent white matter, likely explaining the naming decline. Of the two patients with postsurgical reductions in verbal learning, one declined from average (*z* = 1.2) to low‐average (*z* = −1.6) range after resection of left frontal BOSD; the other declined from superior (*z* = 2.0) to average (*z* = .3) range following resection of left parietal BOSD. Finally, one patient showed a change in visual memory recall following resection of right parietal BOSD; however, their performance remained within the normal limits (presurgical *z* = 1.3, postsurgical *z* = −.6).

## 4 DISCUSSION

### 4.1 Cognitive impacts of early versus late seizure onset

Earlier seizure onset was associated with a greater degree of cognitive dysfunction in adult patients with BOSD, consistent with recent evidence examining malformations of cortical development more broadly.^25^, ^26^ The impact of early life seizures on neurodevelopment is well documented.^27^, ^28^ Animal models have shown that early onset seizures adversely affect the developing brain, hindering maturation of cortical function during critical periods of development.^29^ In children with FCD,^30^ an early age at seizure onset was found to independently contribute to poor cognitive outcomes, regardless of the extent of dysplasia.

Three of the patients in our sample (7%) had an ID, all of whom had a seizure onset before age 2 years. This is consistent with previous findings demonstrating that patients with seizures beginning around infancy are at a higher risk of developing ID.^6^ Furthermore, one study found earlier age at seizure onset during infancy to be associated with poorer neurodevelopmental outcomes, regardless of lesion pathology, seizure frequency, or the type of seizures.^31^ We note the prevalence of ID in our adult study is somewhat lower than that reported in pediatric cohorts. This finding likely reflects a relatively older age at seizure onset among patients seen in our adult epilepsy service.

We found that patients with an older age at first seizure presented a near‐normal cognitive profile. Notably, a majority of those with a later age at onset (>12 years) had parietal BOSD, compared to frontal BOSD. This finding mirrors previous observations.^32^ Despite more than 30% of our sample presenting with parietal BOSD, there was no evidence of visuospatial deficits in the group. An older age at seizure onset was previously linked with a smaller FCD,^32^, ^33^ with FCDs of a more focal nature predictive of better cognitive outcomes than more extensive lesions.^30^ Although we did not compare the size of frontal and parietal BOSDs in this work, we show evidence that a later seizure onset, occurring outside critical periods of neurodevelopment, is associated with less cognitive comorbidity.

### 4.2 Naming and executive dysfunction were the most common deficits at baseline

Neuropsychological assessments were tailored to examine cognitive domains of greatest risk as determined by the individual clinical history and epileptology. Although this could potentially lead to bias in some comparisons, given this targeted approach, the results from our study may be interpreted as representing an upper bound estimate of the cognitive effects. In this context, naming and executive dysfunction emerged as the most frequently impaired domains at baseline, consistent with the pediatric literature.^12^ As a group, one in two patients assessed for naming presented with deficit, with a majority (73% of those exhibiting naming deficit) having a left‐sided BOSD. Naming deficits in these cases may reflect network‐level dysfunction in response to the BOSD in the language‐dominant hemisphere. Our highlighted case of the left temporal BOSD (Figure 5) exemplifies this possibility. Despite their focal nature, BOSDs have been associated with widespread abnormality in white matter fiber tracts.^34^ FCDII lesions have also been implicated in the disruption of local connectivity.^35^ The high prevalence of verbal fluency (a measure of executive function) deficits among patients with a frontal BOSD is unsurprising, with ASM load known to be a contributing factor.^36^

In addition to naming and executive deficits, we also found evidence of dysfunction in other cognitive domains, including processing speed, attention, working memory, and verbal learning. Although deficits in these domains were less commonly seen, they were nonetheless present in approximately 20% of our patients, suggesting careful assessment in clinical practice is warranted.

Given the frontoparietal predilection of BOSD in our sample, we were able to compare groups of patients with frontal and parietal BOSD, highlighting that those with a frontal BOSD tended to perform worse in verbal learning. This is likely secondary to executive contributions to learning, rather than reflective of a primary memory change per se.^37^

### 4.3 Cognition is generally stable postsurgically with favorable seizure outcomes

For the majority of our cases, cognition remained unchanged following targeted resection of BOSD, with only rare occurrences of psychometric decrements. One study reported stable or improved neuropsychological outcomes in 75% of cases following resection of frontal FCDs at 2‐year follow up, noting an unspecified ratio of focal and extensive resections performed in this series.^38^ Postsurgical assessments in the present study were completed at least 3 months postoperatively. Although there was a relatively short duration of follow‐up, we expect patients with long‐term favorable seizure outcomes to experience ongoing stability of cognitive functioning.^39^ Moreover, whereas earlier surgeries in our series may have involved a slightly larger extent of resection (as exemplified in our discussion of a patient who experienced language decline following surgery), our principle has consistently been to favor focal lesionectomy. To the extent that our surgical practice has moved toward smaller resections over time, our data may be seen to represent a conservative representation of surgical cognitive outcomes.

We found group‐level evidence of a modest improvement in processing speed postsurgically, despite the majority (82%) of patients remaining on the same or increased number of ASMs. The improvement in processing speed aligns with the impression of cognitive "brightening" described in a BOSD case study.^16^ Electrophysiological studies have characterized BOSDs as intrinsically epileptogenic lesions, exhibiting almost continuous interictal epileptiform discharges.^40^ We interpret cognitive improvement following BOSD resection as resulting from the removal of "nociferous cortex,"^41^ eliminating interference exerted by the epileptogenic lesion on cortical function beyond the lesion.^42^, ^43^

Even with this postsurgical increment in performance, however, the group average remained below normative expectations. These findings are likely driven by several factors, including the lasting effects of curtailed cognitive development associated with epilepsy,^44^ persisting cerebral changes due to chronic refractory seizures,^34^ and ongoing effects of ASMs.^45^

The absence of significant postsurgical cognitive decline suggests that BOSD tissue is not directly implicated in cognitive functioning and can therefore be safely resected. Previous studies of electrographic functional mapping have suggested minimal, if not an absence of, functionality in BOSDs.^14^ Such findings are consistent with evidence of hypometabolism in the region of BOSD.^12^, ^46^ Reduced functional activity has also been described with the presence of balloon cells,^14^ a histological hallmark of FCDIIB, which is the most common subtype of BOSD.^5^ Safe resection of BOSD located proximal to eloquent cortex has been demonstrated in individual case reports.^10^, ^47^ Our surgical approach involves resecting only the dysplastic tissue, without inclusion of the transmantle band.^9^, ^48^ This reduces the risk of disconnecting fiber tracts and minimizes the impact on neighboring normal cortex.

Approximately 60% of our patient group achieved enduring seizure freedom. Although this figure is somewhat lower than previously reported in pediatric cohorts,^6^, ^8^, ^9^, ^12^ it is comparable to outcomes reported in adult samples.^49^ The discrepancy in seizure control compared to pediatric patients is likely due to the longer duration of epilepsy experienced by adult patients. Early surgery in children often leads to favorable seizure and neurodevelopmental outcomes due to their capacity for neural plasticity and functional recovery.^50^ In comparison, chronic epilepsy in adults can lead to secondary network epilepsy, possibly due to abnormal structural and functional connectivity.^34^, ^51^, ^52^

### 4.4 Limitations

In our cohort of adults with refractory seizures, age at onset appeared as a major determinant of overall cognitive outcome. One question our data cannot address is how outcomes would be affected by early surgical intervention resulting in seizure freedom across the developmental period. This would help disentangle the effects of age at onset per se, versus the combined effects of age at onset and seizure burden. There is evidence that early intervention providing seizure freedom improves cognitive outcomes.^8^, ^50^ The effects of chronic seizure activity on cognitive dysfunction, over and above age at onset, may be measured by a longitudinal comparison between patients with an early seizure onset who either did or did not undergo early surgical intervention.

Furthermore, the cognitive data analyzed in this study were retrospectively collated from clinically directed neuropsychological assessments. Although this approach resulted in an imbalance of sample sizes in some comparisons, it had the advantage of yielding large numbers to explore the cognitive consequences of BOSD. Prospective collection of a uniform dataset, including psychopathology data, seizure frequency over time, and ASM would be useful for systematic characterization of cognition in the setting of BOSD and the localizing value of neuropsychological findings. Nonetheless, our clinically directed assessments were targeted to domains most likely to be affected in each case, arguably maximizing our sensitivity to any cognitive effects in our cohort.

## 5 CONCLUSIONS

Our study of the cognitive features of BOSD‐associated refractory focal epilepsy in an adult epilepsy program was notable for three key observations. First, a younger age at seizure onset was associated with a greater degree of cognitive dysfunction. Second, against a background of mild reductions in speed and attention, deficits were most frequently seen in naming (predominantly in those with left‐sided BOSD) and executive function (predominantly in those with frontal BOSD). Finally, and most importantly for clinical practice, focal lesionectomy of BOSD can achieve high rates of postsurgical seizure freedom with minimal risk of cognitive morbidity.

## AUTHOR CONTRIBUTIONS

Carmen J. Zheng was involved in data acquisition, statistical analysis, data interpretation, manuscript drafting for intellectual content, and final approval of the manuscript and is accountable for all aspects of this work. David Weintrob was involved in data acquisition, data interpretation, revision of the manuscript for intellectual content, and final approval of the manuscript and is accountable for all aspects of this work. Marie O'Shea was involved in data acquisition, data interpretation, revision of the manuscript for intellectual content, and final approval of the manuscript and is accountable for all aspects of this work. Graeme D. Jackson was involved in data interpretation, revision of the manuscript for intellectual content, and final approval of the manuscript and is accountable for all aspects of this work. Chris Tailby was involved in conceptualizing study design, data acquisition, statistical analysis, data interpretation, revision of the manuscript for intellectual content, and final approval of the manuscript and is accountable for all aspects of this work.

## FUNDING INFORMATION

C.J.Z. is supported by an Australian Government Research Training Program Scholarship. C.T. is supported by an NHMRC project grant (APP1157145). G.D.J. and C.T. are supported by the Australian Epilepsy Project which received funding from the Australian Government under the Medical Research Future Fund (Frontier Health and Medical Research Program‐Grant Number RFRHPSI000008). The Florey Institute of Neuroscience and Mental Health acknowledges the strong support from the Victorian Government and in particular the funding from the Operational Infrastructure Support Grant. The authors acknowledge the facilities and scientific and technical assistance of the National Imaging Facility, a National Collaborative Research Infrastructure Strategy (NCRIS) capability.

## CONFLICT OF INTEREST STATEMENT

None of the authors has any conflict of interest to disclose. We confirm that we have read the Journal's position on issues involved in ethical publication and affirm that this report is consistent with those guidelines.

## ETHICS STATEMENT

The study was approved by the Ethics Committee at Austin Health (HREC number H2013/05123; HREC/60011/Austin‐2019; HREC/68372/Austin‐2022), and all participants provided informed written consent.

## Supporting information


**TABLE S1** List of measures and their corresponding cognitive domain included for analysis.
**TABLE S2** Sample sizes across each laterality and focality comparisons for all cognitive measures.

## Data Availability

The data that support the findings of this study are available on request from the corresponding author. The data are not publicly available due to privacy or ethical restrictions.
