# Supplementary material for: Cognition in adults with bottom‐of‐sulcus dysplasia and the consequences of focal resection
Source: Epilepsia. 2026 Feb 5;67(5):2450–62. doi: 10.1002/epi.70116 (PMC13179654; doi:10.1002/epi.70116)
Supplement: Supplementary file 1 — TABLE S1 List of measures and their corresponding cognitive domain included for analysis. TABLE S2 Sample sizes across each laterality and focality comparisons for all cognitive measures. [file EPI-67-2450-s001.docx]

**Supplementary materials**

**Table S1.** **List of measures and their corresponding cognitive domain included for analysis.**

| **Cognitive domain** | **Measure** | **Acronym** |
| --- | --- | --- |
| Processing speed | Digit Symbol Coding | DSCd |
| Attention | Digit span (WAIS) | Dspan |
| Working memory | Trails Making Test (Part B) | TMT-B |
| Verbal fluency | Letter Fluency | FAS |
| Naming | Boston Naming Test | BNT |
| Verbal learning | Rey Auditory Verbal Learning Test (Sum of trials 1-5) | RAVLT1-5 |
| Verbal delayed recall | Rey Auditory Verbal Learning Test (Trial 7) | RAVLT-delay |
| Visual delayed recall | Rey Complex Figure (Delay) | RCF-delay |
| Nonverbal reasoning | Block Design (WAIS/WASI) | BD |
| Verbal reasoning | Similarities (WAIS/WASI) | Simi |
| Background cognitive ability | Weschler Test of Adult Reading | WTAR* |

** In seven patients diagnosed clinically with developmental dyslexia, the WTAR data were excluded as it was considered an invalid estimate of their background cognition.*

**Table S2**. **Sample sizes across each laterality and focality comparisons for all cognitive measures***.*

|  | **Left**  **(n = 17)** | **Right**  **(n = 25)** | **Sig. bias** | **Frontal (n = 22)** | **Parietal (n = 14)** | **Sig. bias** |
| --- | --- | --- | --- | --- | --- | --- |
| Processing speed | 15 | 20 | *ns.* | 20 | 11 | *ns.* |
| Attention | 16 | 19 | *ns.* | 21 | 10 | *ns.* |
| Working memory | 8 | 10 | *ns.* | 14 | 4 | *ns.* |
| Verbal fluency | 12 | 11 | *ns.* | 18 | 3 | *<.01* |
| Naming | 15 | 5 | *<.001* | 13 | 4 | *ns.* |
| Verbal learning | 16 | 21 | *ns.* | 20 | 13 | *ns.* |
| Verbal delayed recall | 14 | 16 | *ns.* | 18 | 9 | *ns.* |
| Visual delayed recall | 6 | 20 | *ns.* | 12 | 10 | *ns.* |
| Non-verbal reasoning | 9 | 22 | *ns.* | 15 | 12 | *ns.* |
| Verbal reasoning | 8 | 9 | *ns.* | 13 | 3 | *ns.* |
| Background cognitive ability | 11 | 10 | *ns.* | 14 | 10 | *ns.* |

*p-*values come from Fisher exact tests; FDR corrected. Grey highlights comparisons subject to a sampling bias and therefore excluded from laterality and/or focality comparisons.
